# 

**Published:** 1862-12

**Authors:** 


### CHICAGO MEDICAL JOURNAL.

**Terms, $2.00 per Annum.**

### CONTENTS OF THE DECEMBER NUMBER.

ORIGINAL COMMUNICATIONS.

PAGE.

Cases of Vesico-Vaginal Fistula. By De Laskie Miller, M. D., Prof. of Obstetrics in Rush Medical College. 667

Rupture of the Uterus—Cæsarean operation—Death in seven days. Reported by L. R. Holmead, M. D., Chicago 670

Remarkable Case of Retention of the Placenta. By J. Drake Harper, M. D., Springfield, Ill 674

A Case. By L. B. Brown, M. D., Iroquois, Ill. 675

Clinical Instruction in the Hospital of Vienna. By E. L. Holmes, M. D. Chicago 676

SELECTED.

Diphtheria. Extract Proceedings Philadelphia County Medical Society. Jas. M. Corse. M. D., Chairman Committee 682

Clinical Researches upon Auscultation of the Head. By Henry Roger, Prof. Fac. Med., Paris 693

Substance of a Clinical Lecture on a Case of Embolon of the Femoral Artery, etc. Under Care of Dr. Pitman and Mr. Prescott Hewett 701

Is Alcohol Food? By Thos. Inman, M. D., London, Physician to the London Royal Infirmary 704

Treatment of Scarlatina. By T. Hillier, M. D 707

The Chlorine and Milk Treatment of Scarlet Fever and the Typhoid Fevers. By Conway T. Edwards, M. R. C. S. E. 712

EDITORIAL AND MISCELLANEOUS 719

